# *Plasmodium* Infections in Natural Populations of *Anolis sagrei* Reflect Tolerance Rather Than Susceptibility

**DOI:** 10.1093/icb/icx044

**Published:** 2017-07-07

**Authors:** Camille Bonneaud, Irem Sepil, Lena Wilfert, Ryan Calsbeek

**Affiliations:** * Centre for Ecology and Conservation, University of Exeter, Penryn, TR10 9EF, UK; † Department of Zoology, University of Oxford, Oxford, OX1 3PS, UK; ‡ Department of Biological Sciences, Dartmouth College, New Hampshire, Hanover, NH 03755, USA

## Abstract

Parasites can represent formidable selection pressures for hosts, but the cost of infection is sometimes difficult to demonstrate in natural populations. While parasite exploitation strategies may, in some instances, actually inflict low costs on their hosts, the response of hosts to infection is also likely to determine whether or not these costs can be detected. Indeed, costs of infection may be obscured if infected individuals in the wild are those that are the most tolerant, rather than the most susceptible, to infection. Here we test this hypothesis in two natural populations of *Anolis sagrei*, one of the most common anole lizard of the Bahamas. *Plasmodium* parasites were detected in > 7% of individuals and belonged to two distinct clades: *P. mexicanum* and *P. floriensis*. Infected individuals displayed greater body condition than non-infected ones and we found no association between infection status, stamina, and survival to the end of the breeding season. Furthermore, we found no significant difference in the immuno-competence (measured as a response to phytohemagglutinin challenge) of infected versus non-infected individuals. Taken together, our results suggest that the infected individuals that are caught in the wild are those most able to withstand the cost of the infection and that susceptible, infected individuals have been removed from the population (i.e., through disease-induced mortality). This study highlights the need for caution when interpreting estimates of infection costs in natural populations, as costs may appear low either when parasites exploitation strategies truly inflict low costs on their hosts or when those costs are so high that susceptible hosts are removed from the population.

## Introduction

Harboring parasites is energetically costly to hosts, not only because they exploit host resources, but also because they cause damage to host tissues and activate costly immune responses (Sheldon and Verhulst 1996; Bonneaud et al. 2012). Access to limited resources means that any reallocation of energy to parasite proliferation, tissue repair or immune activation will divert it away from other fitness-associated traits, such as physical activity, thereby giving rise to the physiological constraints underlying life-history trade-offs (e.g., between survival and reproduction) (Bonneaud et al. 2003; van der Most et al. 2011). While evidence for energetic costs of infection is accumulating (Eraud et al. 2005; Bonneaud et al. 2016), the impact of infection on other fitness-associated traits remains difficult to demonstrate in natural populations (Knowles et al. 2009). One key reason is that it is unclear whether infection in wild-caught individuals reflects increased susceptibility or heightened tolerance to parasites. In both of these cases, wild-caught individuals that are not infected will comprise of resistant, as well as unexposed hosts. However, whether infection reflects susceptibility or tolerance will have consequences for the pool of infected individuals, since susceptible individuals that are infected will be removed from the population (i.e., through disease-induced mortality) in the latter, but not in the former case. Because energy should become limiting primarily in infections of resistant and susceptible hosts (due to protective immune activity and pathogenesis, respectively; Bonneaud et al. 2012), and less so of tolerant individuals (Råberg et al. 2007), trade-offs resulting from infection may therefore not always be apparent in the wild.

*Plasmodium* parasites, which are transmitted to vertebrate hosts by haematophagous dipteran vectors during blood meals, have the potential to cause high levels of morbidity and mortality in natural populations (Van Riper et al. 1986). Pathogenesis is caused primarily by the high metabolic demands of *Plasmodium* proliferation, hemoglobin catabolism for the biosynthesis of parasite amino acids, and massive lysis of infected erythrocytes, all of which give rise to shortages of oxygen and glucose necessary for cellular metabolism in host tissues (Roth 1990; Mackintosh et al. 2004; Olszewski et al. 2009). Consequently, *Plasmodium* infections have been shown to be associated with substantial metabolic complications in a range of organisms, in part, due to a mismatch between oxygen supplies and requirements of host tissues (Li et al. 2008; Olszewski and Llinas 2011). For instance, in humans, severe malaria is marked by low blood glucose levels (hypoglycaemia) and build-up of lactate in the body (lactic acidosis) due to increased anaerobic glycolysis (Planche et al. 2005). Western Fence Lizards (*Sceloporus occidentalis*) infected with *Plasmodium mexicanum*, displayed a 25% reduction in hemoglobin concentration and 30% increase in oxygen consumption following physical exertion relative to uninfected individuals, evidencing similar increased reliance on anaerobic metabolism and greater costs of recovery (Scholnick et al. 2010). *Plasmodium* infection also increased the cost of recovery following physical activity in *S. occidentalis*, with infected lizards displaying heightened blood glucose and lactate levels relative to non-infected ones (Scholnick et al. 2012). Such metabolic complications are expected to impair the physical activity of *Plasmodium*-infected hosts and, accordingly, classical symptoms of severe malaria in humans include muscle aches, contractures, fatigue, and weakness (Miller et al. 1989).

*Plasmodium* infections have been associated with cardiac dysfunction and shown to have detrimental effects on skeletal muscles in both humans (Miller et al. 1989; Nguah et al. 2012; Yeo et al. 2013; Marrelli and Brotto 2016) and animals (Carmona et al. 1996; Vuong et al. 1999; Brotto et al. 2005; Scholnick et al. 2012). While such pathogenic effects are thought to be primarily driven by tissue hypoxia (Yeo et al. 2013), investigation of the contractile function and biochemical properties of the skeletal muscles of mice infected with *P. berghei* revealed direct effects on the contractile machinery itself (Brotto et al. 2005). Indeed, the leg muscles of infected mice displayed a significant loss of essential contractile proteins that was likely responsible for a 50% decrease in contractile force, heightened fatigue, and lower recovery from fatigue. Atlantic canary (*Serinus canaria*) infected with *P. cathemerium* exhibited similar skeletal muscle compromise, with marked alterations in their contractile and sarcotubular systems (Carmona et al. 1996). Such muscle cell damage is thought to result from the inflammatory and oxidative stress triggered during malaria (Callahan et al. 2001; Clark and Cowden 2003; Pabon et al. 2003). Despite measurable effects on muscle function in humans and animals in the laboratory, there remains considerable variation in estimates of the impact of *Plasmodium* on physical activity in natural populations (Merino et al. 2000; Schall and Pearson 2000; Knowles et al. 2010).

Impacts of *Plasmodium* infection on activity in the wild have been investigated as direct measures of locomotor capacity, as well as indirectly by evaluating effects on higher-level phenotypes mediated by physical performance (e.g., reproductive effort). For instance, natural *Plasmodium* infections were found associated with reduced stamina in both western fence and rainbow (*Agama agama*) lizards (Schall 1990). However, there was no association between *Plasmodium* infection status and sprint speed in western fence lizards (Schall 1990), or locomotor activity in Spiny lizards (*Sceloporus jarrovii*) (Halliday et al. 2014). *Plasmodium* infection nevertheless impacted social interactions in western fence lizards, with infected males being more often socially submissive, less socially active, and less able to maintain territories and defend access to females (Schall and Dearing 1987; Schall and Sarni 1987). *Plasmodium* infections have also been shown to have mix effects on reproductive success in the wild. Female blue tits (*Cyanistes caeruleus*) that were infected and treated with an anti-malarial drug displayed increased hatching success, provisioning rates and fledging success relative to infected females that were untreated (Knowles et al. 2010). In contrast, the same population of blue tits also exhibited a positive association between reproductive effort (measured as clutch size) and parasitaemia (Knowles et al. 2011), and no association was reported between infection status and reproductive performance in red-billed gulls (*Larus scopulinus*) (Cloutier et al. 2011). The association between *Plasmodium* infection status and physical activity is likely to be, in large part, dependent on the actual cost of the parasite’s exploitation strategy. But greater virulence may not necessarily be associated with greater measurable costs if virulence is so high that infected individuals that are susceptible are removed from the population, thus biasing the pool of infected individuals towards those that are able to withstand the cost of infection.

We investigated whether infection with *Plasmodium* signals increased susceptibility or heightened tolerance in natural populations of *Anolis sagrei* lizards. To do so, we screened wild-caught lizards for *Plasmodium* parasites and examined links between infection status, body condition, locomotor performance (stamina), and survival to the end of the breeding season. We predicted that, if infection signals increased susceptibility to *Plasmodium* (hereafter: the susceptibility hypothesis), infected lizards should exhibit reduced body condition, locomotor performance, and survival relative to non-infected ones. Conversely, a lack of association or positive associations between infection status and those traits would support the hypothesis that, under natural conditions, wild-caught infected individuals are those that are able to tolerate the costs of infection (hereafter: the tolerance hypothesis). In addition, we predicted that the immuno-competence of infected individuals would be lower than that of non-infected individuals if infection reflects greater susceptibility (Navarro et al. 2003). To test this additional prediction, we challenged all individuals with phytohemagglutinin (PHA), which stimulates the infiltration and/or proliferation of various immune cells, including T lymphocytes (Licastro et al. 1993; Martin et al. 2006), and is hence commonly used in eco-immunology to estimate cell-mediated immunity (for e.g., Gonzalez et al. 1999; Martin et al. 2003; Svensson et al. 2001; Mugabo et al. 2015; Bowers et al. 2014).

## Methods

### Study system and field methods

The brown anole, *A. sagrei*, is a small (40–70 mm snout-vent-length; SVL) semi-arboreal lizard, and is one of the most common anoles in the Bahamas (Losos 2009). We studied wild populations of *A. sagrei* at two sites of the Bahamas: Regatta Point on the large island of Great Exuma (23°30′25.1″N 75°45′58.3″W) and Stocking Island (23°32′N 75°46′W), a ∼1 km^2^ island <2 km offshore. We captured a total of 343 individuals, 130 from Regatta Point (66 females and 64 males) and 207 from Stocking Island (52 females and 155 males) during spring (May–June) 2005. Upon capture, we measured body mass (nearest g) and assigned each individual with a unique four-color combination of elastomer markings, which were injected into the underside of the hind- and forelimbs. Blood was drawn from the postorbital sinus and stored in PBS/EDTA buffer at −20 °C, and we measured immune-competence using a PHA assay (see below). All lizards were then released back to their site of capture and a subset of them (from Regatta Point only) was recaptured 2 weeks later to measure running endurance.

Most lizards (∼90%; Cox and Calsbeek 2010) in our study population mature and die in a single year. We therefore estimated fitness as survival from initial capture (sub-adulthood) in late May–early June to our population censuses conducted during late September–early October. This 4-month period accounts for survival to maturity and to the end of the first breeding season. Lizards that we did not recapture were considered to have died; this is a reasonable assumption since emigration from islands is extremely rare, except perhaps during hurricanes (Calsbeek and Smith 2003), of which none occurred during this study. Moreover, although the majority of surviving lizards were recaptured within the first 2 days of our census, we searched an additional 3 weeks to ensure the recapture of every marked lizard. Censuses continued until two consecutive days with no new recaptured individuals. In total, we recaptured 108 individuals, including 47 on Regatta Point (19 females and 26 males) and 60 on Stocking Island (12 females and 48 males).

### Screening for *Plasmodium* infection

DNA was extracted for all samples from whole blood following a DNeasy kit protocol (Qiagen, Valencia, CA, USA). We used primers and methods described in Perkins and Schall (2002) to detect *Haemoproteus* and *Plasmodium* parasites, which are euprotista belonging to the phylum apicomplexa. The PCR products were run on 2% agarose gels and stained with ethidium bromide for UV detection. Negative results were confirmed by repeated PCR. The PCR products were purified using a MinElute Qiagen^®^ kit following manufacturer’s instructions. We identified lineages by sequencing the fragments (BigDye (R) version 1.1 sequencing kit, Applied Biosystems) on an ABI PRISM 3100 (TM) sequencing robot (Applied Biosystems). Distinct sequences found several times in independent PCRs, either within a same individual or in several different individuals, were considered to be verified (V). Unique sequences, which only differed from verified sequences by one nucleotide, were also found. However, a single nucleotide divergence may be attributed to a *Taq* polymerase incorporation error during amplification or to another type of PCR error (jumping PCR, heteroduplex artifact) and these haplotypes are therefore considered non-verified (NV). Sequences are deposited in GenBank^TM^ with the following accession numbers DQ846851-DQ846861 and DQ986492-DQ986495.

### Immune response

*In vivo* cell-mediated immune response was assessed using a PHA assay (Goto et al. 1978). Because males are larger than females, we challenged males with 0.20 mg PHA in 0.02 ml PBS and females with 0.10 mg PHA in 0.01 ml PBS, injected in the left hind-foot pad. We injected the same volume of PBS in the right hind-foot pad as a control. We recorded the thickness of each footpad with dial-calipers (±0.01 mm) at the site of PHA injection, before and again 24 h following injection. We assessed the intensity of the response to PHA as the difference in swelling between the PHA-injected and the control footpad. Swelling was measured in a total of 194 individuals, including 77 from Regatta Point (39 females and 38 males) and 118 from Stocking Island (9 females and 109 males). All individuals were released back at their site of capture following immune measure.

### Stamina

Individuals on Regatta Point were re-captured after 2 weeks to ensure full recovery from immune measurements. Stamina was then measured by running lizards to exhaustion on an electrical treadmill (0.4km/h) (Perry et al. 2004). Because anoles do not run well on level surfaces (Perry et al. 2004), we set the treadmill at a 20° incline. We motivated lizards to run by manually tapping the hind limb. Lizards were considered to have run to exhaustion after three failed attempts to induce running, and/or the loss of the lizard’s natural righting response. Stamina was measured as the time to exhaustion (in seconds) in a total of 127 individuals from Regatta Point only (64 females and 63 males).

### Phylogenetic and statistical analyses

**Fig. 1:**
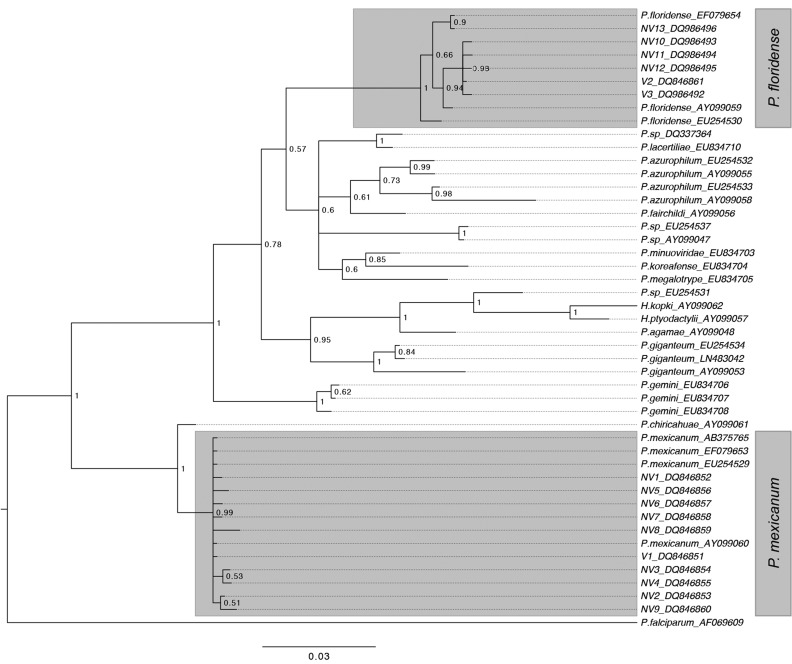
Phylogenetic tree 15 *Plasmodium* isolates found in *A. sagrei* based on *cytochrome b* sequences. The phylogeny of the *cytochrome b* gene was reconstructed using a Bayesian approach. Sequences from known lizard malaria parasites were included for comparison, and human *P. falciparum* was used as an out-group. V1 belongs to the monophyletic group of *P. mexicanum*, while V2 and V3 verified lineages belonged to the monophyletic group of *P. floridense*. GenBank accession numbers of all sequences are indicated. Numbers on interior branches indicate Bayesian support.

The phylogeny of the isolates was reconstructed using a Bayesian approach in MrBayes v.3.2.6 (Huelsenbeck and Ronquist 2001) and includes reptilian malaria isolates available on Genbank, as well as *Plasmodium falciparum*, which is used as an outgroup. The phylogeny is based on 598 bp of the *cytB* gene. Genbank accession numbers are included in the tree annotation (Fig. 1). The tree was reconstructed using a gamma-distributed, site-specific, general time-reversible model, with parameters estimated from the data during the analysis. We ran two runs of two chains for 20,000,000 MCMC generations, sampling trees every 20,000 generations. The tree was then plotted using Figtree v1.4.2 (http://tree.bio.ed.ac.uk/software/figtree/).

**Fig. 2:**
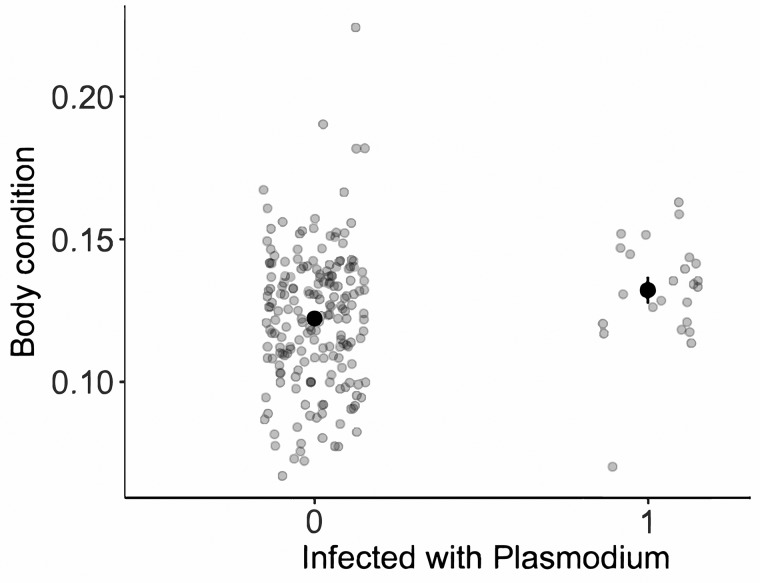
Association between *Plasmodium* infection status and body condition in male *A. sagrei*. The darker symbols show the predicted means and se, and the lighter symbols show the raw values.

**Fig. 3:**
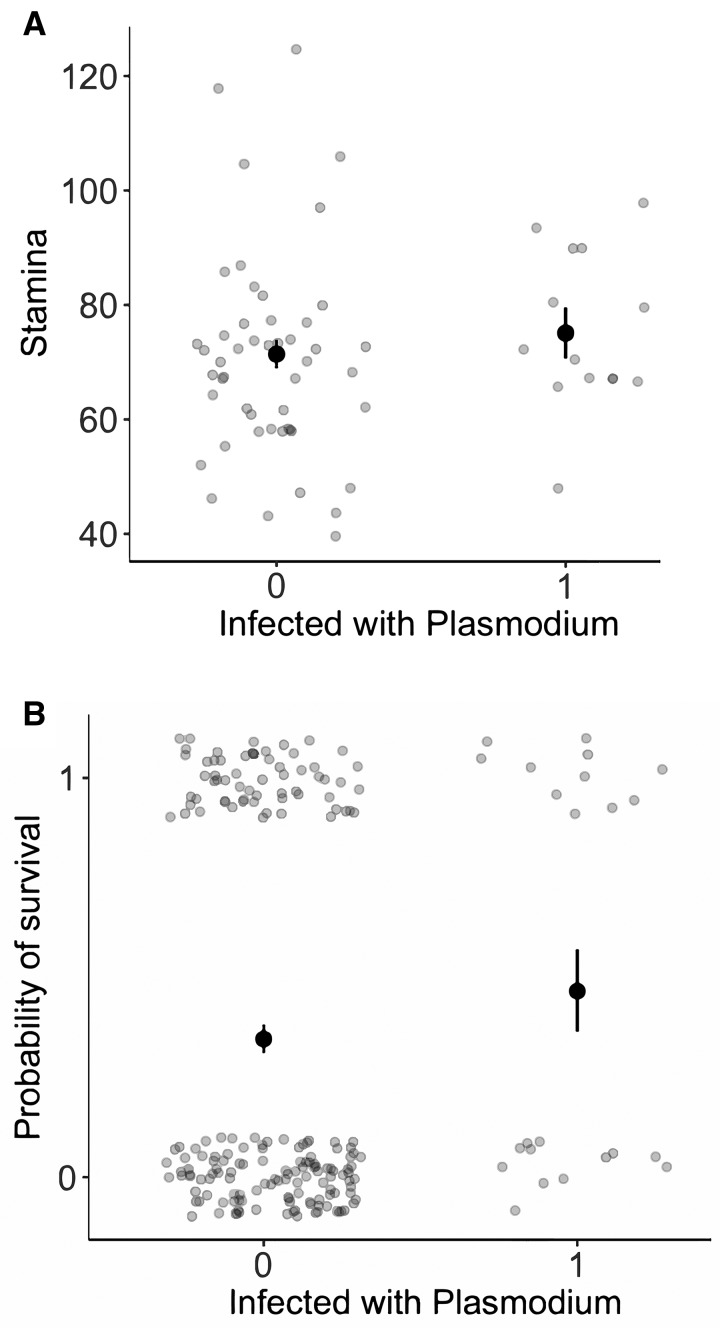
Association between *Plasmodium* infection status and (**A**) stamina (in s) and (**B**) survival to the next breeding season in male *A. sagrei*. The darker symbols show the predicted means and se, and the lighter symbols show the raw values. In (B), note the dispersion of observations around 0 (no survival) and 1 (survived) to improve the visualization of results.

**Fig. 4:**
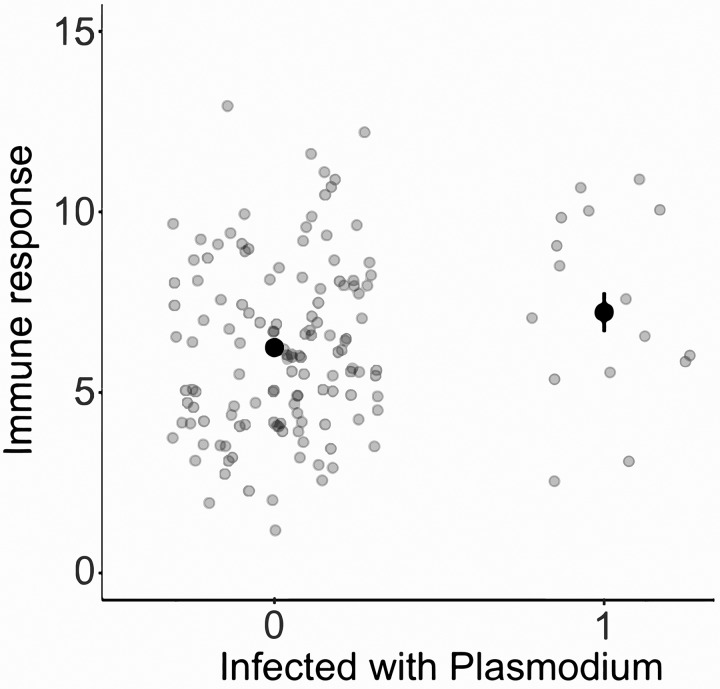
Association between *Plasmodium* infection status and immune swelling (in mm) to PHA in male *A. sagrei*. The darker symbols show the predicted means and se, and the lighter symbols show the raw values.

All statistical analyses were conducted in R 3.3.2 (R Core Team 2016). Out of the 25 individuals that tested positive for *Plasmodium* infection, only one was female. As a result, all analyses were done on males only. First, we tested whether body condition was affected by infection status using linear regressions with body condition as the response variable and with infection status as the explanatory term. Body condition was calculated as (body mass/SVL^2^) with body mass in milligrams and SVL in millimeters; by doing so, our analysis controls for any differences in SVL that are generated by differences in age and/or growth rate. To test for differences in stamina as a function of infection status, we then used a linear regression with stamina as the response variable and with infection status and body mass as the explanatory terms. We investigated whether individuals experience different survival probability depending on their infection status using a logistic regression with survival to the end of the breeding season as the response variable and with infection status and body mass as the explanatory terms. Finally, we modelled differences in immune response using a linear regression that included immune swelling as the response variable and with infection status and body mass as the explanatory variables. Figures 2–4 were made using the package ggplot2 (Wickham 2009).

## Results

Out of 337 individuals, 25 (7.4%) were infected with *Plasmodium* lineages, with prevalence differing significantly between sites and reaching 12% on Regatta Point and 5% on Stocking Island (*χ*^2^ = 4.3, df = 1, *P* = 0.04). Of the 25 infected lizards, only one was a female from Stocking Island. Out of the 24 males infected, 15 (63%) were from Regatta Point and nine (38%) from Stocking Island. Sequencing *Plasmodium* infections in all 25 infected individuals yielded 15 unique sequences (597 bp), only three of which were verified mitochondrial malaria lineages (Fig. 1). All sequences belonged to two well-supported monophyletic clusters of *Plasmodium* lineages, with V1 and NV1-9 belonging to the clade containing *P. mexicanum* and V2, V3, and NV10-13 belonging to the clade containing *P. floridense* group. No individual was found to be co-infected with *P. mexicanum* and *P. floridense*.

We found no compelling support for the susceptibility hypothesis: in no case was there a negative association between infection status and host traits, and any non-significant associations were all in a positive direction (Table 1). Males that were infected were found to be in significantly better body condition than non-infected males (linear regression; infection status: *t*_1,215_ = 2.0, *P* = 0.04; *R*^2^ = 0.02; Fig. 2). There was no effect of infection status on male stamina (linear regression; infection status: *t*_1,60_ = 0.8, *P* = 0.46; body mass: *t*_1,60_ = 2.2, *P* < 0.04; *R*^2^ = 0.09; Fig. 3A). Similarly, there was no association between survival to the next breeding season and infection status (logistic regression; infection status: *z*_1,202_ = 1.1, *P* = 0.26, relative odds ratio = 1.7 (CI = 0.68–4.0); body mass: *z*_1,202_ = 1.2, *P* = 0.24, relative odds ratio = 1.0 (CI = 0.99–1.0); Fig 3B). Finally, immune swelling in response to PHA tended to be higher in infected males, but this effect was not significant (linear regression; infection status: *t*_1,143_ = 1.73, *P* = 0.09; body mass: *t*_1,143_ = 5.3, *P* < 0.001; site: *t*_1,143_ = 0.6, *P* = 0.54; *R*^2^ = 0.19; Fig. 4).

**Table 1:** Model estimates and standard errors for each of four models testing the association between infection status with *Plasmodium* parasites and host traits

Response variable	Explanatory variables	Estimate	SE
Body condition	Infection status	0.01	0.005
Stamina	Infection status	3.81	5.09
	Body mass	0.04	0.02
Survival	Infection status	0.50	0.45
	Body mass	0.001	0.001
Immune response	Infection status	1.02	0.60
	Body mass	0.007	0.001

## Discussion

*Plasmodium* infections were detected in >7% of wild-caught *A. sagrei*, with prevalence ranging from 12% on the main island of Great Exuma (Regatta Point) to 5% on the more remote Stocking Island. Lizards were infected either with *P. mexicanum* or with *P. floridense*, and both *Plasmodium* clades were found at both sites. Despite demonstrated costs of *Plasmodium* infection in other taxa in both laboratory and natural settings, we found that infected male had higher body condition than non-infected ones. Furthermore, infection with *Plasmodium* was not associated with reduced stamina, survival, or immune swelling to PHA and any trend was in a positive direction in contrast to the predictions of the susceptibility hypothesis (Table 1). Although these trends were not significant in the opposite direction to those expected under the susceptibility hypothesis, power analyses revealed that considerably more individuals would be required to obtain significance for each parameter tested (e.g., 463 for stamina and 322 for immune response). Our results are therefore consistent with the prediction that wild-caught lizards infected with *Plasmodium* are tolerant, rather than susceptible, to the parasite.

While studies on humans and laboratory animals demonstrate measurable costs of *Plasmodium* infections with detrimental consequences on host traits (e.g., body condition, physical activity), evidence of such effects in natural populations remains mixed (Merino et al. 2000; Schall and Pearson 2000; Knowles et al. 2010). For several years now, this has fueled debate as to whether or not *Plasmodium* infections are actually truly costly in the wild (Asghar et al. 2011). Comparisons across host populations and *Plasmodium* lineages reveal that costs of infection can, in fact, vary markedly. For example, the widespread population declines and extinctions suffered by the Hawaiian avifauna as a result of the introduction of *P. relictum* attests to the fact that infections may be more costly in recently exposed hosts (Van Riper et al. 1986). Furthermore, the fitness consequences of infection may also vary depending on the *Plasmodium* lineage involved. Lesser Kestrels (*Falco naumanni*) displayed reduced fledging numbers only when infected with one of two *Plasmodium* lineages detected in this species (Ortego et al. 2008). Interestingly, while on the whole correlative studies estimating the cost of *Plasmodium* infection remain inconclusive, experimental manipulations of *Plasmodium* infection through the administration of anti-malarial medication demonstrate that chronic infections with *Plasmodium* can indeed have significant effects on host fitness (Marzal et al. 2005; Knowles et al. 2010). As a result, the absence of measurable cost to *Plasmodium* infection in natural populations does not necessarily imply that there is no cost *per se*. Rather our ability to estimate this cost will depend on whether we are able to sample all the individuals of the population that have been infected, or whether our sample includes only the subset of individuals that can sustain the costs of infection.

Tolerance is the ability to limit the damages caused by infection for a given parasite load (Råberg et al. 2009). In order words, while tolerant individuals are not able to control their parasite burden, they are able to diminish the associated pathogenic effects. Accordingly, an experimental infection of five strains of mice with *P. chabaudi* revealed measurable differences in tolerance to infection, with the most tolerant mice strains exhibiting reduced loss of both body mass and red blood cells relative to the least tolerant ones (Råberg et al. 2007). Tolerance therefore has the potential to lessen, if not erase, the cost of infection in wild populations. The lack of associations between stamina, survival and *Plasmodium* infection status in our populations of *A. sagrei* evidence an absence of measurable costs of infection. Furthermore, we found that, in fact, infected individuals were in better body condition than non-infected ones. Taken together, these results suggest that wild-caught infected *A. sagrei* encompass the individuals that are able to bear the cost of infection by *Plasmodium* parasites, rather than those that are the most susceptible to infection. While we cannot fully exclude the possibility that infected *A. sagrei* are those that are quantitatively resistant to infection (i.e., able to limit parasite growth; Gandon and Michalakis 2000; Sepil et al. 2013) rather than tolerant, the absence of measurable costs of infection expected as a result of immune activity suggests that this is unlikely to be the case.

That *Plasmodium*-infected lizards are the most tolerant rather than the most susceptible is further supported by the fact that infected individuals did not display reduced immuno-competence relative to non-infected ones. The link between infection status and measures of immune capability (i.e., immuno-competence) is still debated and questions remain as to whether measures of immunity mirror an individual’s health (i.e., whether or not it is currently infected), or whether these measures are indicative of the individuals’ ability to control and clear parasites (reviewed in Biard et al. 2015). The PHA-induced swelling test stimulates the infiltration and/or proliferation of various immune cells, including T lymphocytes (Licastro et al. 1993; Martin et al. 2006), and is hence commonly used in eco-immunology to estimate cell-mediated immunity (e.g., Bowers et al. 2014; Mugabo et al. 2015). Links between the response to PHA and infection status with various parasites is mixed, with some studies showing positive associations and others reporting negative ones (Biard et al. 2015). However, the one study that has tested links with haemosporidian parasites (genus *Haemoproteus*) found that infected house sparrows (*Passer domesticus*) had lower PHA responses and that individuals in better body condition had stronger immune responses to PHA than individuals in lower condition (Navarro et al. 2003). That our study shows a trend for infected *A. sagrei* to display increased immune responses to PHA relative to non-infected ones is therefore more consistent with the hypothesis that infected lizards are tolerant rather than susceptible to infection. Experimental work is, however, now required to fully understand the link between infection status with hemosporidians (including *Plasmodium*) and response to PHA.

Our study highlights the need to take into account the complexity of host–parasite co-evolutionary interactions when evaluating the costs of infection. Virulence, which is strictly defined as parasite-induced host mortality but which can be more broadly thought of as the fitness cost of infection to the host, is a product of both parasite and host behavior and hence an outcome of their interaction (Poulin and Combes 1999; Alizon et al. 2009; Bull and Lauring 2014). As a result, we will only gain a complete understanding of disease virulence and the intensity of parasite-driven selection, if we measure infection costs in an unbiased sample of the host population. However, we are at risk of under-estimating those costs when virulence is such that all susceptible hosts are removed from the population (i.e., through mortality) and that the only infected individuals remaining are the tolerant ones.
